# Seeking research questions from implementers: considerations for leveraging ground actors research needs in the fight against malaria in West Africa

**DOI:** 10.1186/s12936-021-03634-0

**Published:** 2021-03-08

**Authors:** Tete S. Amouh, Saidou Malam Ekoye, Césaire D. Ahanhanzo, Tinga Robert Guiguemdé, Issiaka Sombié

**Affiliations:** 1 West African Health Organization, 175, Avenue Ouezzin Coulibaly, BP: 153 Bobo Dioulasso 01, Burkina Faso; 2 World Health Organization, Niamey, Niger; 3 National Academy of Science of Burkina Faso, Ouagadougou, Burkina Faso

**Keywords:** Malaria, Research collaboration, West Africa, Implementation science

## Abstract

**Background:**

To strengthen the fight against malaria, it is imperative to identify weaknesses and possible solutions in order to improve programmes implementation. This study reports experiences gained from collaboration between decision-makers and researchers from a World Bank project (Malaria and Neglected Tropical Diseases in the Sahel, SM/NTD). The objectives of this paper were to identify bottlenecks in malaria programme implementation as well as related research questions they bring up.

**Methods:**

Questionnaire addressed to National Malaria Control Programme managers and prioritization workshops were used as a medium to identify research questions. The bottlenecks in malaria programme implementation were identified in seven thematic areas namely governance, human resources, drugs, service provision, use of prevention methods, monitoring and evaluation (M and E), and public support or buy-in. The first five priority questions were: (1) compliance with drug doses on the second and third days during the seasonal chemoprevention (SMC) campaigns, (2) the contribution of community-based distributors to the management of severe cases of malaria in children under 5 years, (3) the SMC efficacy, (4) artemisinin-based combination therapy (ACT) tolerance and efficacy according to existing guidelines, and (5) the quality of malaria control at all levels of the health system.

**Results and conclusion:**

This work showed the effectiveness of collaboration between implementers, programmes managers, and researchers in identifying research questions. The responses to these identified research questions of this study may contribute to improving the implementation of malaria control programmes across African countries.

## Background

More often than not, local social determinants of health are hidden or overlooked by funding agencies, institutional researchers, and health systems, yet they may be slowing down or reducing programme implementation and impact. In addition, many of the physical constraints that impede the regular and effective delivery of health interventions to those who need them are much more pronounced in Low-to-Middle-Income Countries (LMIC) than in high income countries [1]. Hence bridging the research divide between the obligations of donors and researchers or field actors are of paramount importance in the fight against diseases, especially malaria in Africa.

Indeed, despite recent trends showing a reduction in malaria mortality rates in Africa generally, and in the region in particular [2], mortality rates remain high in countries such as Nigeria, Burkina Faso, Niger, Mali, Côte d’Ivoire, Ghana, and Guinea [3]. This raise questions about the effectiveness of the fight against malaria in these countries. An approach to identify these weaknesses, opportunities, and the search for solutions to reduce these preventable mortalities can improve control and bring these countries nearer to their pre-elimination targets.

This study reports experiences gained from collaborations between decision-makers and researchers, a part of a project undertaken in the framework of the regional World Bank funded project titled: Malaria and Neglected Tropical Diseases in the Sahel (SM/NTD)[4]. The objectives of this World Bank project were to identify bottlenecks in programme implementation as well as the related research questions they present. The results of this study can help funding agencies to prioritize and fund research activities in a bid to improve the fight against malaria in the beneficiary countries.

## Methods

Local and primary providers along with malaria programme managers were approached in the 15 countries of the Economic Community of West African States (ECOWAS), including eight French-speaking, five English-speaking and two Portuguese-speaking countries using two approaches, namely: a questionnaire survey and regional validation workshops were held as part of the activities of the SM/NTD project. This study only focuses on malaria.

### The questionnaire survey

The questionnaire translated into the three official languages of ECOWAS, namely English, French and Portuguese, were sent to the malaria control programmes managers in the last semester of trimester of 2016. The questionnaires were divided into two (2) parts: (i) Bottlenecks in malaria programme implementation (ii) Priority research questions on malaria programme. Each part covered seven thematic areas: governance, human resources, drugs, service provision, use of prevention methods, monitoring and evaluation (M and E), and public support or buy-in. Questionnaires were thereafter sent through electronic messages (Email) to National Malaria Control Programme (NMCP) managers/coordinators of the 15 ECOWAS countries, with instructions on how to fill the questionnaires.

Follow up actions were undertaken for two months to obtain a satisfactory response rate. Even though the countries have similarities as well as differences regarding their governance structure, epidemiological trends, and public health research, the authors conducted an initial in-depth analysis country by country. They then split bottlenecks recorded and questions into the seven “ECOWAS regional topics of interest” according to the thematic areas mentioned above. This analysis was then presented at the ECOWAS malaria regional workshop.

### Organization of the regional validation workshop

A two-day regional workshop was organized in Bamako by the West African Health Organization (WAHO), the ECOWAS specialized institution dealing with health concerns, such as malaria as well as NTDs programmes in West Africa. Participants included malaria programme managers, NTDs programme managers, Directors of Public Health of the various Ministries, monitoring/evaluation officers, countries project management unit coordinators, technical and financial partners (World Health Organization, World Bank, Helen Keller International, Malaria Consortium, Catholic Relief Services, and several WAHO officers). The results of the questionnaire survey were analysed, presented, and discussed in two plenary and two breakout sessions.

The first breakout session on malaria was organized with two groups of 12 people each. The breakout sessions brought together malaria programme coordinators, researchers, partners, and the regional project team. These teams worked to validate the research problems and questions. The two breakout teams were tasked to review, complete the problems, prioritize, and justify the research questions. The two breakout teams reviewed the survey results using individual and average ratings. For the first plenary session, each group was instructed to choose the top 20 research questions. On the second plenary session, the representative of each group shared the top 20 research questions they have selected. The questions selected by the participants clarified some research questions and allowed to finalize list related to malaria management.

The second breakout session focused on individual countries’ participants. The participants worked together to select three priority research questions from the list of questions established during the plenary session. Representatives of each country were asked to prioritize the three questions they selected and to address them over a three-year (2017, 2018 and 2019) period. Finally, a third plenary session allowed each country’s team to share their key questions to address and prepare a plan for the subsequent years, 2017, 2018 and 2020.

The workshop was moderated by a expert in parasitology and malaria research from West Africa and at the end, the moderator reformulated some of the questions and justifications in accordance with the adopted guidelines.

## Results

Out of the 15 ECOWAS countries, 11 (Benin, Burkina Faso, Côte d’Ivoire, Gambia, Ghana, Guinea, Mali, Niger, Nigeria, Senegal, and Togo) responded to questions related to malaria and Table 1 shows the bottlenecks identified by programme managers/coordinators according to the seven areas addressed in the questionnaire.

**Table 1:** Bottlenecks in MALARIA programme implementation in West Africa

Theme	Malaria
Governance	Difficulty in accessing information from some partners Inadequate collaboration with the private, para-public and religious communities at the district and regional levels Inadequate capacity for management and coordination of control at the regional and district levels Inefficiency in the implementation of SMC strategy? Low availability of funds to support programme management activities Weak cross-border collaboration and networking Difficulty in delimiting partners' intervention zones
Human resources	Lack of capacity, mobility Lack of adequate motivation Lack of a career plan Inadequate capacity to design at the programme level
Drugs	Out of stock (management) Incomplete and poor data quality Low storage capacity (districts, sites) Low follow-up of efficiency and resistance Shortage of SP Poor adverse reaction reporting Weak quality control Counterfeit medicines
Service provision	Absence of initial treatment prior to transfer of severe malaria case What happened to tablets/drug left to the parents after 1st SMC distribution? Insufficient directly observed SP treatment Organizational deficiency in ANC Low coverage of pregnant women with IPT2/3 at ANC Insufficient funding for LLIN EC campaign and operational costs Poor compliance with guidelines Insufficient coverage of services
Use of prevention methods	Non-optimal use of LLINs, IPT Absence of insecticides for impregnating curtains Population's poor perceptions of the use of LLINs Low utilization/late attendance of ANC for IPT/SP Insufficient mechanism for monitoring home dosing Insufficient coverage of services
Monitoring and evaluation	Difficulty in collecting community and private data Availability, quality, completeness, timeliness and archiving of data Inadequate supervision Insufficient dissemination of research results Lack of data on mortality due to malaria Weak monitoring system
Public support or buy-in	Poor adoption of behaviors in favour of the fight against malaria Insufficiency, reluctance, non-adherence to the 2 home doses of the SMC by some parents Fixed strategy disallowed by some parents, door-to-door preference as for national immunization days

In all seven thematic areas, there were challenges that limited the effectiveness of programme implementation. In the area of governance, issues of coordination and collaboration with partners in the field came up as well as weaknesses in the managerial capacity at the regional and district levels. The human resources problems identified were mainly related to capacity, competence, motivation of community health workers, weaknesses in research skills and capacity of programme actors. There were also difficulties in the area of management of medicine logistics, from ordering commodities to the distribution to patients, and also difficulties in pharmacovigilance. In service provision thematic area, difficulties of direct observed treatment were pointed out, especially for the second and third day doses of SMC, while for prevention, low uptake of vector control measures, the absence of insecticides for the impregnation of protective materials, and the low use of protective means were the major problems mentioned. In terms of monitoring and evaluation, challenges of access to quality data, especially from the community level, and inadequacy in the dissemination of research results were reported.

Finally, in terms of public support, refusal or reluctance to participate in mass drug distribution during SMC campaigns, failure in adopting preventive measures and behaviour were reported by programme managers.

Table 2 presents the 21 priority issues as ranked by the participants by thematic areas.

**Table 2:** Priority research questions on malaria specific programme areas, in West Africa

Rank	Research questions	Justifications
Gouvernance		
3	What is the most effective strategy (door-to-door, cluster sites…) for seasonal malaria chemo-prevention?	There is a need to find adequate strategies for a good implementation of SMC campaigns, taking into account local constraints
5	What is the quality of malaria management at different levels of the health system?	The insufficient quality data on the management of malaria in health facilities and poor estimation of malaria cases, is often a challenge for decision making for planning and implantation
14	What part NICT (new information and communication technology) has in the management of quality data?	Due to the low level of completeness, promptitude and archiving, there is need to look into the use of technology
17	What system of collaboration should be put in place between Ministry of Health and other partners?	Insufficient coordination of activities have been recorded across the board
Human resources		
2	What is the contribution of Community Drug Distributors in the management of severe malaria cases in children under five years of age as part of pre-transfer treatment?	There is often inadequate treatment (pre-transfer treatment) of severe malaria before the transfer to better facility. This delays treatment on arrival and recovery
16	Can CHWs contribute to improving the supervised administration of IPT-2 and IPT-3?	There is a need to know how programmes can improve the low coverage of IPT-2 and IPT-3
Drug/malaria medicines		
4	How effective and tolerable is ACT (artemisinin-based combination therapy) under actual conditions of use?	More data are needed on the efficacy and tolerability of ACT under actual conditions of use
6	What are the adverse effects of anti-malarial drugs (artemisinin-based combination, SP-AQ)	More data are needed on the pharmacovigilance of anti-malarial drugs in mass drug distribution: toxicological effects especially hepatic
13	How effective are the medicines used in traditional medicine in the of malaria case management at the community level?	The high use of traditional medicine by the population and the low collaboration between traditional and modern medicine should be explored
21	What is the comparative advantage of the dihydroartemisinin (DHA)-piperazine combination over SP-AQ?	Due to its side effects and limitations, programmes should look also for an alternative to SP-AQ as part of the SMC
Service		
11	What is the quality of the supply/commodities?	There are counterfeit medicines in circulation, leading to treatment failure and difficult diagnosis
10	What is the performance of input supply chain systems?	Commodities stock-outs, insufficient rational management of medicines, are often recorded
Prevention		
7	How effective are insecticides in long-lasting insecticidal nets (LLINs) in vector control?	After few years of use, there is a lack of updated efficacy data on insecticides used in LLINs
9	What are the factors for non-use of LLINs in vector control?	Managers and health workers have noticed a low level of public support for the use of LLINs
18	What is the level of use and effectiveness of indoor residual spraying for malaria prevention at the community level?	There are insufficient data on its IRS efficiency
19	How effective is the mosquito repellent soap developed in Burkina Faso as part of a multicenter study?	Research should contribute to find new alternative means of prevention to LLINs
20	What is the feasibility and effectiveness of impregnating nets with two insecticides?	With few years of the current tools, there is a need to develop of new means or tool of vector control in order to limit the spread of resistance of malaria vectors
Monitoring and evaluation		
1	What are the factors that influence adherence to day 2 and day 3 doses during SMC?	The effectiveness of the ongoing strategy is not fully documented leading to insufficient data and monitoring of compliance with 2nd and 3rd doses in the community
12	What is the diagnostic performance of RDTs at the facility level?	Little is known about the performance of real-world/real-time RDTs (rapid diagnostic test)
15	What is the level of morbidity and mortality attributable to malaria?	There is a need to evaluate the impact of control programmes
Populations adherence/buy-in		
8	What are the communication channels, supports and strategies that induce the most behavior change?	Behavioral change at community and individual level has been slow

The first five priority questions were related to (1) compliance with drug doses for the second and third days of SMC campaigns, (2) contribution of community-based distributors to severe malaria cases management in children under 5 year, (3) SMC efficacy, (4) ACT efficacy and tolerance of ACT under current guidelines, and (5) quality of malaria cases management at all health system levels.

The prevention theme was ranked first with five questions, followed by the governance and medicines with four questions, and the monitoring and evaluation (M and E) with three questions. The five prevention questions were related to the effectiveness and non-use of long-lasting insecticidal nets LLIN), the level of use and effectiveness of indoor residual spray (IRS), conducting a study on the effectiveness of mosquito soap, and the possibility of using two insecticides to impregnate nets. Governance issues were related to the best strategy for the implementation of SMC campaigns, quality of malaria case management at all health system levels, the place of information and communication technology in data quality management and the type of collaboration framework between the Ministry of Health and partners. For the medicines theme, the four priority questions focused on the efficacy and tolerance of ACT under current guidelines, the side effects of ACT, the efficacy of traditional medicines and the comparative advantage of the dihydroartemisinin (DHA)-piperazine combination over sulfadoxine–pyrimethamine (SP) + amodiaquine (SP-AQ). Regarding M and E, questions related to adherence for the second and third dose during seasonal chemoprevention campaigns, the performance and use of rapid diagnostic tests. In terms of human resources, the two priority issues were related to the contribution of community health workers in severe malaria case management and the supervision of the second and third doses during SMC campaigns. At the service delivery level, both issues were related to the quality and performance of the drug supply and management chains. Finally, in terms of public support, the questions were related to communication channels, media and strategies to ensure behavior change. Table 3 shows the three priority issues identified by the three project country teams, namely Burkina, Mali and Niger.

**Table 3:** Countries programming priority research questions on malaria

Countries	Priority issues malaria
Burkina Faso	What is the most efficient strategy (door-to-door, cluster sites…) for seasonal malaria chemo-prevention (SMC)?
	What are the factors that influence adherence to day 2 and day 3 doses during SMC?
	What is the role of CHW in the supervised administration of IPT2 and IPT3 in pregnant women?
Mali	What are the factors that influence adherence to Day 2 and Day 3 doses during SMC campaign?
	What are the adverse toxicological effects of multiple administration of anti-malarial drugs, SP-AQ in children?
	What is the impact of communication interventions on the adoption of behaviors favourable to the fight against malaria?
Niger	What are the factors that influence adherence to day 2 and day 3 doses during SMC campaign?
	What is the therapeutic effectiveness of QA + SP?
	What is the role of CHW in the supervised administration of IPT2 and IPT3 in pregnant women?

It was noted that the research questions related to factors influencing adherence to second day (day 2) and third day (day 3) doses of SMC were highlighted as first priority by Mali and Niger participants. The same questions were flagged as second priority by Burkina Faso participants. It was followed by questions related to the contribution of community health workers in the supervision of the second and third doses of drug during the SMC campaigns in Burkina and Niger. The final ones were in order of importance, the questions on the most efficient strategy in the implementation of SMC in Burkina Faso, the toxicological effects of administering multiple doses of SP-AQ in children, the impact of communication interventions in 2nd and 3rd days in Mali, and the therapeutic effectiveness of SP-AQ in Niger.

## Discussion

This work allowed the identification of key challenges limiting the implementation of NMCP in West Africa. Priority research themes in malaria has helped to highlight similarities between countries regarding malaria control programmes. Most of the bottlenecks highlighted by this work are related to the poor coordination and collaboration with partners, the skills and motivation of community health workers responsible for drug distribution during mass treatment. In addition, the bottlenecks were also due to weaknesses in the supply and distribution chain, poor use of prevention measures, difficulties in accessing quality data, especially at community level, and the population buy-in and acceptance of strategies. The problems that have emerged in malaria control programmes in this work have already been reported by some authors in Africa [5–11]. The results are consistent with previous reports regarding difficulties related to malaria management. With regards to research questions, there is an urgent need for information sharing on SMC strategy and implementation to allow effective malaria control and eradication. Community health workers and the general population must be mobilized and involved in the fight against malaria. The research questions on SMC seem consistent with reports that showed that there is little implementation research in malaria eradication programmes in the three countries, Burkina, Mali and Niger. The majority of the research conducted related to SMC in West Africa, were clinical studies on the effectiveness of the malaria programmes strategies and these studies [6, 9, 12–14] provide some answers to operational questions, however, more research is needed in malaria control across different countries.

This work was designed to assess the implementation, operational bottlenecks, and success of public health practice in West Africa. Furthermore, this study evaluated the relationship between programme implementing actors, African researchers, and the constraints they face in their respective countries. Local researchers should work in coordination with programme actors to address local problems. The New Partnership for Africa’s Development (NEPAD)’s Consolidated Plan of Action 2005–2014 (CPA) and the Science Technology Innovation Strategy 2024 [15] attempted to ensure the continent’s collective commitment towards an innovation-led knowledge development. Science and technology must be incorporated in different local strategies to address African problems, as evidence based research interventions are often required by donors and governments to improve the implementation of public health activities.

The landscape of global health keeps changing because new innovations and new discoveries are being implemented in interventions against emerging diseases (infectious and non-communicable). New discoveries either technically or “process, are not easy to incorporate in public health interventions since they require drawing new strategic plans and other logistics. Hassan [16] has reported the difficult conditions in developing countries regarding research expertise and the lack of evidence-based research required to inform interventions, this makes developing countries inexistent when it comes to research in the context of globalization. Southern partners (especially developing countries) have generally identical social burdens and environmental conditions [17], therefore, there is a need to increase intra-regional collaboration.

After the identification of research priority questions, the project funded some research activities taking into account these priorities. For instance, a regional research was commissioned in the three countries related to the factors that influence adherence to day 2 and day 3 doses during SMC. The research started in 2018 and the primary result will be obtained in 2020. The overall results will be validated in the countries and published in 2021. Still at the regional level, a second research was conducted to analyse all communication interventions and impact on malaria programmes. Currently the preliminary results on the mapping of communication interventions are being finalized and the impact study is ongoing. Between 2018 and 2020, a lot quality assurance sampling survey (LQAS) was conducted in Burkina Faso and Mali in order to analyse the performance of the SMC campaign. Further research work is needed to on research questions not addressed in this study. In Mali, three studies were conducted including the prevalence of *Plasmodium falciparum* carriage rate in the SMC implementing areas; on adverse toxicological effects of multiple administration of SP-AQ, and on potential factors affecting adherence to mass drug administration (MDA) in nomadic population.

## Conclusion

This study showed the effectiveness of collaboration between policy makers and researchers in identifying and funding research needed to improve malaria control programmes. The research questions identified can be adapted to other ongoing research to improve malaria control in different countries.

## Data Availability

Data supporting the results reported in the manuscript article can be found at West African Health Organization (WAHO) archives and in the references provided in the manuscript.

## Abbreviations

ACT: Artemisinin-based combination therapy
ANC: Antenatal care
CHW: Community health worker
CPA: Consolidated plan of action
DHA: Dihydroartemisinin
ECOWAS: The Economic Community of West African States
IPT: Intermittent preventive treatment
LMIC: Low-to-middle-income countries
LLIN: Long-lasting insecticidal nets
LQAS: Lot quality assurance sampling survey
MDA: Mass drug administration
M and E: Monitoring and evaluation
NEPAD: The new partnership for Africa’s development
NMCP: National malaria control program
NICT: New information and communication technology
SM/NTD: Malaria and neglected tropical diseases in the Sahel
SP-AQ: Sulfadoxine-pyrimethamine + amodiaquine
RDT: Rapid diagnostic test
SMC: Seasonal malaria chemoprevention
WAHO: West African Health organization
